# Supplementary material for: The clinical performance of ultra-low-dose shoulder CT scans: The assessment on image and physical 3D printing models
Source: PLoS One. 2022 Sep 26;17(9):e0275297. doi: 10.1371/journal.pone.0275297 (PMC9512178; doi:10.1371/journal.pone.0275297)
Supplement: S1 File — (PDF) [file pone.0275297.s002.pdf]

Ethics Committee of Guangdong Provincial Hospital of Chinese Medicine Approval Notice

Approval Number：广东省中医院伦理委员会BF2019-030-01

Review date	Preliminary examination: February 22, 2019
Review place	2005-2006 (long conference room), 20th floor, research building, Guangdong Hospital of traditional Chinese Medicine
Clinical research approval	--
Clinical research project	Application of low dose CT scanning in 3D printing image acquisition before fracture operation
Review documents	1. The initial review application form includes a statement of economic interests of the study<br>2. Study protocol (version No. / date: 01 / 20190221)<br>3. Informed consent form provided to the subject (including research introduction and signature page) (version No. / date: 01 / 20190221)<br>4. Resume of main researchers<br>5. GCP training certificate of main researcher<br>6. List of professional team members
Sponsor / task issuing unit	Guangdong Medical Research Fund Project
Clinical research unit	Zhuhai Hospital of Guangdong Hospital of traditional Chinese Medicine
Principal investigator	Mengqiang Xiao
Ethical review method	Meeting review
Review committee	Xing Zeng , LAN Cheng ,Xiaohui Qiu , Teng Huang ,Qingyi Huang , Zehuai Wen , Bojian Chen ,Yong Li , Haoxi Feng
Review comments	According to the code for the quality management of clinical trials of drugs, the code for the quality management of clinical trials of medical devices and the guiding principles for the ethical review of clinical trials of drugs issued by the State Food and drug administration, the measures for the ethical review of biomedical research involving human beings and the measures for the management of stem cell clinical research (Trial) issued by the health and Family Planning Commission, The code for the construction of ethical review platform for clinical research of traditional Chinese Medicine issued by the State Administration of traditional Chinese medicine, as well as the ethical principles of the declaration of Helsinki of the World Medical Association and the international ethical guide for biomedical research of human body issued by the International Committee of Medical Sciences, have been reviewed by this ethics committee, We agree to conduct clinical research in accordance with the above clinical research plan and the above reviewed documents.

Ethics Committee statement	This approval document will be filed in each central institution and its ethics committee. If you have different opinions on the feasibility of the scheme in this institution (including the qualification and experience of researchers, equipment and conditions, etc.), please contact this ethics committee in time.<br>If the project is suspended / terminated in advance / the clinical study is completed, or serious adverse events and unexpected adverse events affecting the risk benefit ratio of the study occur, please report to the ethics committee in time. In case of any modification of the clinical research protocol and informed consent and the replacement of the main investigator, the ethics committee shall be notified in time and implemented after review and approval. Any violation of the protocol that affects the subjects' willingness to participate in the study shall be reported in time. Please submit the research progress / conclusion report one month before the expected follow-up review date to facilitate the follow-up review of the project.<br>Research projects involving China's human genetic resources and requiring approval must be approved by the China human genetic resources management office [illegible]
Validity of approval	From February 22, 2019 to February 22, 2021<br>Follow up review frequency 12 months<br>Expected review date February 22, 2020
contact number	020-81887233 to 35943, contact: Xiaoyan Li
Signature of chairman / Vice Chairman	

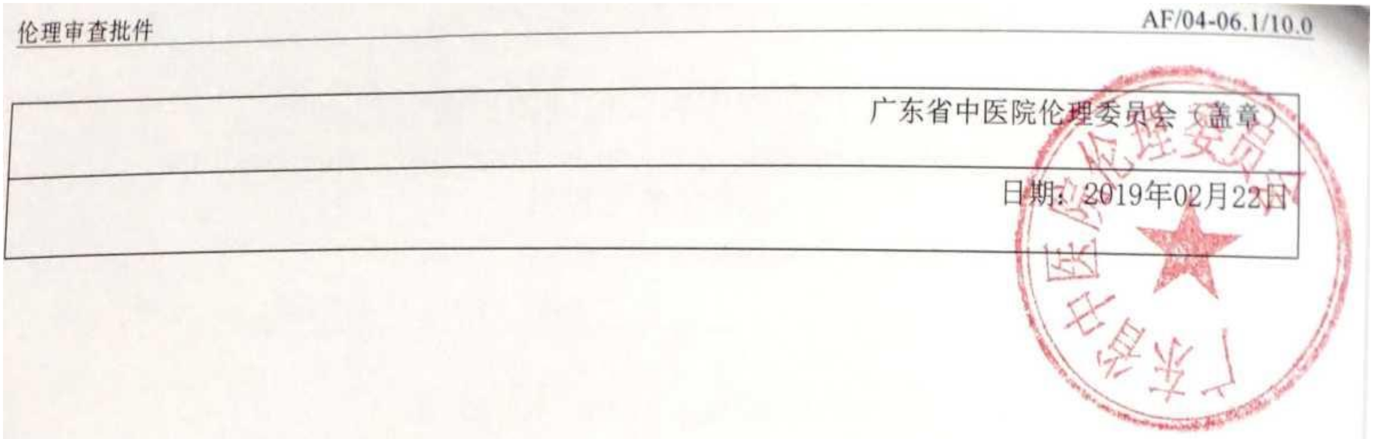
伦理审查批件 AF/04-06.1/10.0

广东省中医院伦理委员会（盖章）

日期：2019年02月22日

AF/02-06.1/23.1

广东省中医院伦理委员会

Ethics Committee of Guangdong Provincial Hospital ofChinese Medicine

会议签到表

Sign-in Sheet of Full IEC Meeting

日期：2019 年 02 月 22 日 审议项目：低剂量 CT 扫描在骨折术前 3D 打印图像采集的应用研究 伦理委员会到会委员签名：

姓名	职务	性别	专业情况	签名
–刘军~~	主任委员	男	中医骨伤科学、伦理学、管理学	请假
曾星	副主任委员	女	I 期临床、分子生物学、伦理学	
刘旭生	副主任委员	男	中医内科学	请低
程兰	委员	女	中医妇科学	
丘小惠	委员	女	中药制剂学	f
温泽淮	委员	男	中医内科学、循证与临床研究方法学	
魏琳	委员	女	护理学	请假
黄腾	委员	男	中医儿科学	，生七
黄庆仪	委员	女	中医内科学	
罗懿妮	委员	女	临床药学、中药学	
陈伯健	委员	男	中医骨伤科	
林同香	委员	男	细胞生物学	请假
李立凯	委员	男	法律代表（非医药学）	请敢
李泳	委员	男	法律代表（非医药学）	
冯昊禧	委员	男	社区代表（非医药学）	
