# Supplementary material for: The clinical performance of ultra-low-dose shoulder CT scans: The assessment on image and physical 3D printing models
Source: PLoS One. 2022 Sep 26;17(9):e0275297. doi: 10.1371/journal.pone.0275297 (PMC9512178; doi:10.1371/journal.pone.0275297)
Supplement: Attachment — Submitted filename: PONE-D-22-07553_Response letter.docx [file pone.0275297.s004.docx]

Dear Dr. Bianchi,

Thank you very much for your decision letter and advice on our manuscript (Manuscript No. PONE-D-22-07553) entitled “The clinical performance of ultra-low-dose shoulder CT scans: the assessment on image and physical 3D printing models”. We also thank the reviewers for the constructive comments and suggestions. We have revised the manuscript accordingly, and all amendments are indicated by red font in the revised manuscript. In addition, our point-by-point responses to the comments are listed below this letter.

This revised manuscript has been edited and proofread by *Medjaden* Inc..

We hope that our revised manuscript is now acceptable for publication in your journal and look forward to hearing from you soon.

With best wishes,

Yours sincerely,

Mengqiang Xiao

First of all, we would like to express our sincere gratitude to the reviewers for their constructive and positive comments.

Reviewer #2: This manuscript describes the clinical performance of ultra-low-dose shoulder CT scans: the assessment on image and physical 3D printing models.
I have two comments.
(1). Material and Methods: patient selection
How about the pathological fracture patient? Were these patients included in the study? The authors did not answer this question.
Response: Patients with pathological fractures were not included in the study. The following sentences were added to the Materials and Methods section.

“Exclusion criteria were as follows: less than 18 years of age, pregnancy, patients who refused to participate in the study, and patients with osteoporosis (bone density: lumbar spine and/or hip joint T-score < -2.5) or pathological fractures.”

(2). line 143-147:” Displaced fractures refer to avulsion fractures caused by a sudden and violent contraction of a muscle or ligament, resulting in a long-diameter low than 5 mm wide bone piece. Non-displaced fractures are defined as no angulation or shortening, less than 2 mm wide fracture line and/or less than 1 mm displacement of the bone cortex [21].”
This description will still confuse the reader. Please clarify the definition. Displaced fracture is not equal to the avulsion fracture. How much displacement (distance) or angulation (angle) was defined as displaced fracture in this study? Please clarify the definition.

Response: The following sentences were added.

Line 143-147: “Non-displaced fractures are defined as having no angulation or shortening, a fracture line less than 2 mm wide and/or less than 1 mm displacement of the bone cortex. Displaced fractures are defined as having angulation or shortening, a fracture line more than 2 mm wide and/or more than 1 mm displacement of the bone cortex. Avulsion fractures caused by a sudden and violent contraction of a muscle or ligament, were grouped into non-displaced fractures or displaced fractures when the fracture had a long-diameter ≤ 5 mm or > 5 mm wide bone piece, respectively [21].”
